# Demethylation of Alkali Lignin with Halogen Acids and Its Application to Phenolic Resins

**DOI:** 10.3390/polym11111771

**Published:** 2019-10-28

**Authors:** Hao Wang, Thomas L. Eberhardt, Chunpeng Wang, Shishuai Gao, Hui Pan

**Affiliations:** 1 Jiangsu Co-Innovation Center for Efficient Processing and Utilization of Forest Resources, Nanjing Forestry University, 159# Longpan Road, Nanjing 210037, China; wy782455481@163.com; 2 College of Chemical Engineering, Nanjing Forestry University, 159# Longpan Road, Nanjing 210037, China; 3 Forest Products Laboratory, USDA, One Gifford Pinchot Drive, Madison, WI 53726, USA; thomas.l.eberhardt@usda.gov; 4 Institute of Chemical Industry of Forestry Products, Chinese Academy of Forestry, Nanjing 210042, China; wangcpg@163.com (C.W.); gaoshishuai1006@163.com (S.G.)

**Keywords:** alkali lignin, demethylation, halogen acids, phenolic resin

## Abstract

Lignin, a byproduct from the chemical processing of lignocellulosic biomass, is a polyphenolic compound that has potential as a partial phenol substitute in phenolic adhesive formulations. In this study, HBr and HI were used as reagents to demethylate an alkali lignin (AL) to increase its hydroxyl content and thereby enhance its reactivity for the preparation of phenolic resins. Analyses by FT-IR, ^1^H-NMR and 2D-NMR(HSQC) demonstrated both a decrease in methoxyl groups and an increase in hydroxyl groups for each demethylated lignin (DL). In addition, the molar amounts of phenolic hydroxyls, determined by ^1^H-NMR, increased to 0.67 mmol/g for the HI-DL, and 0.64 mmol/g for the HBr-DL, from 0.52 mmol/g for the AL. These results showed that HI, a stronger nucleophilic reagent than HBr, provided a higher degree of AL demethylation. Lignin-containing resins, prepared by copolymerization, met the bonding strength standard for exterior plywood with DL used to replace as much as 50 wt.% of phenol. The increased hydroxyl contents resulting from the lignin demethylations also imparted faster cure times for the lignin-containing resins and lower formaldehyde emissions. Altogether, the stronger nucleophilicity of HI, compared to HBr, impacted the degree of lignin demethylation, and carried through to measurable differences the thermal properties and performance of the lignin-containing PF resins.

## 1. Introduction

Lignin is the phenypropanoid polymer that encrusts the hemicelluloses and cellulose in plant cell walls, and binds the individual cells together. It comprises 15 to 35% of lignocellulosic biomass, and represents the most abundant renewable aromatic substance found in nature [1]. To date, most lignin that is separated from wood fibers by chemical pulping is used for its fuel value; less than 2% of the approximately 70 million tons of lignin produced by the paper industry is used for alternative applications (e.g., concrete additives, surfactants) [1]. Regarding the conversion of lignocellulosic biomass to fuels and chemicals in a biorefinery, efforts to improve the economics of such operations have emphasized the development of higher value uses for the residual lignins; however, the chemical heterogeneity and relatively low reactivity of these lignins also lends them to be used as fuel [2]. Therefore, the development of modified lignins with improved properties (e.g., physical, thermal) and/or reactivity seems to be a rational start to increase the use of this sustainable biopolymer [3,4].

Phenol formaldehyde (PF) resin is a thermosetting adhesive with wide applications for exterior-grade construction panels including oriented strand board (OSB), plywood, and containerboards, owing to its high bonding strength, resistance to moisture, and temperature stability. PF resin is generally synthesized from petroleum-derived phenol, formaldehyde and either acidic (novolacs) or alkaline (resoles) catalysts. Lignin has been investigated as a sustainable alternative to petroleum-derived phenol in PF resin production due to its structural similarities [5]. Several avenues have been explored in the preparation of lignin-based phenolic resins: lignin directly used as a filler; lignin used with the addition of filler agents; and lignin used after chemical modification [6]. Only a small amount of unmodified lignin, at an additive level of 5–10% of the resin weight, can be applied in the preparation of phenolic resin adhesives due to its aforementioned chemical heterogeneity and relatively low reactivity [7,8].

Chemical modifications, including phenolation and methylolation, have been used as effective means to improve the reactivity of lignin for the further application in phenolic resin synthesis [9]. Phenolation has been used to increase the number of phenolic groups. Alonso et al. [10]. found that lignin can be phenolated at 120 °C for 160 min using oxalic acid as a catalyst; the resultant phenolated lignin was used to replace up to 30% of the phenol in a phenolic resin. Through methylolation, hydroxymethyl functional groups were added to the lignin structure [11]. Hydroxymethylated lignin was prepared by Vázquez et al. [9], and the modified lignin was used to replace the phenol in a phenolic resin, also achieving a lignin substitution of up to 30%.

Alternatively, the removal of lignin functional groups can also improve its reactivity in the formulation of phenolic resins. Specifically, through demethylation, the lignin chemical structure is modified by removing one or two methoxyl groups from ortho positions to the phenolic hydroxyl. Demethylations have been carried out using various nucleophiles and the resultant modified lignins used in phenolic resin synthesis [12,13,14,15]. Recently, Li et al. [13] prepared demethylated lignin with sodium sulfite for a phenolic resin; bonding strength still met the Chinese national standard (GB/T 9846-2015) for exterior-grade plywood panels (0.7 MPa) when one half of the phenol was replaced with demethylated lignin.

Since complete demethylation of lignin is achieved with the very strong nucleophile hydroiodic acid (HI), this halogen acid is used for quantitative determinations of lignin methoxyl contents [16,17]. Recently, Sawamura et al. [18] demethylated a synthetic lignin with three different reagents, 1-dodecanethiol, HI, and iodocyclohexane. The two iodine-based reagents provided the best results; however, recondensation reactions were observed with iodocylohexane. Hydrobromic acid (HBr), a weaker halogen acid than HI, has also been used to demethylate lignin; this reaction was not carried out with the halogen acid alone, but included hexadecyltributylphophonium bromide [19]. In the current study, we hypothesized that HBr, being a weaker nucleophile than HI, would likely generate a modified lignin, but with perhaps a lower degree of demethylation. If indeed correct, it remained to be determined whether differences in the degree of demethylation extended to detectible differences reactivity for the generation of phenolic resins. Accordingly, these two halogen acids, HI and HBr, were used in a side-by-side comparison as the nucleophilic reagents for lignin demethylation. The resulting demethylated lignins were characterized by FT-IR, ^1^H-NMR and 2D-NMR(HSQC). The demethylated lignins were then used to prepare phenolic resin adhesives which were then characterized by FT-IR and ^13^C-NMR. The mechanical properties of the lignin phenol formaldehyde (LPF) resins were investigated and compared with that of the normal PF resin.

## 2. Materials and Methods

### 2.1. Materials

Alkali lignin (AL), product number L0082, was purchased from TCI Shanghai Co., Ltd. (Shanghai, China). The lignin was washed with aqueous 2 M hydrochloride acid and filtered in a sand core filter funnel, and then repeatedly washed with excess water until neutral. The filtered lignin was dried overnight in a vacuum oven at 40 °C and stored in a tightly sealed container. HBr (48 wt.%), HI (48 wt.%), phenol, formaldehyde (37 wt.%), ether, pyridine, acetic anhydride, *N,N*-dimethylformamide (DMF) and sodium hydroxide were AR grade reagents and purchased from Sinopharm Chemical Reagent Co. Ltd. (Shanghai, China).

### 2.2. Demethylation of Alkali Lignin

Two halogen acids, HBr and HI (both 48 wt.% aqueous) were used as the reagents for the demethylation of an AL. The demethylation method was adapted from the literature [19] and optimized as follows: reaction temperature was 130 °C; reaction time was 12 h, and catalyst:AL ratio was 4:1 (*w*:*w*), for each halogen acid (HI or HBr). In brief, 500 mg AL was dissolved in 2.5 mL DMF in a 50 mL pressure tube followed by the addition of 2 g 48% aqueous halogen acid. The mixture was then heated at 130 °C with continuous stirring at 500 rotations/min by magnetic stirring. At the end of the reaction time, the pressure tube was removed from heat and cooled under ambient conditions. Then the mixture was added dropwise into 250 mL of 2 M aqueous HCl solution to precipitate the demethylated lignin. The precipitate was vigorously stirred for 3 h before filtering, and washing with excess of water until neutral. The wet residue was then dried overnight in a vacuum oven at 40 °C. The dried demethylated lignin, as a solid powder, was purified by suspending in 20 mL diethyl ether, and vigorously stirring for 10 min. The demethylated lignin was recovered in a sand core filter funnel and dried under vacuum overnight at 40 °C. Demethylated lignins, using either HBr or HI as reagent, were labeled as HBr-DL and HI-DL, respectively.

### 2.3. Characterization of Alkali Lignin and Demethylated Lignins

The FT-IR analyses of the AL and demethylated lignin (DL) samples were performed on a Nicolet 380 spectrometer (Thermo Fisher Scientific, Shanghai, China). All lignin samples were combined with KBr powder and pressed into pellets. Scans were made in the range of 4000 cm^−1^ to 500 cm^−1^, a resolution of 4 cm^−1^, and at room temperature. At least 3 spectra were acquired from each lignin sample.

Prior to the ^1^H-NMR measurement, AL and DL samples were acetylated with acetic anhydride and pyridine to convert hydroxyl groups to acetoxy groups. In brief, a lignin sample (200 mg) was dissolved in a 1:1 (*v*:*v*) acetic anhydride:pyridine mixture (8 mL) and stirred for 48 h at room temperature. Then, the reaction solution was added dropwise into cold water to precipitate the acetylated lignins that were collected by filtering the suspension through a sand core funnel. The resulting solid product was exhaustively washed with water to ensure the complete removal of acetic acid. The final product was dried overnight in a vacuum oven at 40 °C.

Acetylated lignins were dissolved in DMSO-*d*6 and analyzed by ^1^H-NMR using an AVANCE III HD 600MHz spectrometer (Bruker Biospin, Bern, Switzerland). A small amount of *p*-nitrobenzaldehyde (10 mg) was added as an internal standard for each sample to compare the changes of methoxyl content before and after modification. The changes in methoxyl and hydroxyl contents were quantified using the ^1^H-NMR spectra.

For the quantitative 2D-NMR heteronuclear single quantum coherence (HSQC) spectra, approximately 60 mg of lignin samples was dissolved in 0.6 mL DMSO-*d*6 and analyzed on an AVANCE III HD 600MHz spectrometer. The relative content of functional groups in lignin is obtained by the integral ratio of their corresponding characteristic peaks in the HSQC spectra.

### 2.4. Preparation of Lignin-Containing Phenolic Resins

Phenol formaldehyde (PF) and lignin phenol formaldehyde (LPF) adhesives were synthesized in a 250 mL three-neck round bottom flask equipped with a reflux condenser and a thermometer. The molar ratio of phenol to formaldehyde was 1:2.2. In the first step, 32 g phenol, 40 g formaldehyde (37 wt.%), and 6.7 g NaOH solution (50 wt.%) were mixed in the flask; the mixture was heated at 60 °C for 30 min. In the second step, 10 g formaldehyde (37 wt.%) and 2 g NaOH (50 wt.%) were added to the flask and the reaction temperature was raised to 70 °C and then held there for 30 min. In the third step, 9.5 g of formaldehyde (37 wt.%), 2 g NaOH (50 wt.%), and 35 g water were added to the reaction flask and the reaction temperature was further increased to 90 °C and then held there for 60 min. The viscosity of the PF resin was closely monitored during the third stage of the synthesis. The PF resin was rapidly cooled to 40 °C when the viscosity reached 150 ± 20 mPa.s.

The preparation of alkali lignin phenol formaldehyde (ALPF) and demethylated lignin phenol formaldehyde (DLPF) resins was similar to the procedure of PF, the exception being that part of the phenol was substituted by AL, HBr-DL, or HI-DL. The obtained ALPF resins with 10%, 30%, and 50% (*w*/*w*) AL substitution were labeled as L10, L30, and L50, respectively. Similarly, the obtained DLPF resins with 10%, 30%, 50% HBr-DL or HI-DL substitution were labeled as B10, B30, and B50, or I10, I30, and I50, respectively.

### 2.5. Characterization of the Lignin-Containing Phenolic Resins

All resin samples were freeze-dried, and then milled to 200 mesh particle size for analysis by FT-IR and liquid-state ^13^C-NMR spectroscopies (Bruker Biospin, Bern, Switzerland). FT-IR analysis of PF, ALPF, and DLPF samples were collected as described above. Samples of resins (50 mg) were dissolved in DMSO-*d*6 for liquid-state ^13^C-NMR analysis using an AVANCE III HD 600MHz spectrometer; all spectra were recorded at room temperature with a relaxation delay of 8 s over 800 scans.

DSC analysis of PF, ALPF, and DLPF resins were determined using a DSC 60A (Shimadzu Corporation, Tokyo, Japan). A 5–10 mg sample was added to an aluminum sample pan and heated at a rate of 10 °C/min from ambient temperature to 150 °C under N_2_ atmosphere at a flow rate of 40 mL/min.

Thermal gravimetric analysis (TGA) was performed on a TA-60H (Shimadzu Corporation, Tokyo, Japan) under N_2_ atmosphere over a temperature range of 25−800 °C, a heating rate of 10 °C/min, and a flow rate of 40 mL/min.

The gel time of phenolic resins was determined according to the Chinese National Standard (GB/T 14074.3-2006). A 5 g sample of the resin was placed in a test tube and maintained in an oil bath at 130 °C. Next, a thin wire spring was used to manually mix the sample until gelation occurred.

### 2.6. Preparation of Plywood

Wheat flour 10–20% by weight to phenolic resin was used in the adhesive mix as an extender. Samples of three-layer plywood (400 mm × 400 mm × 4.5 mm) were prepared from eucalyptus veneers. Both sides of the middle veneer were coated with 24 g (150 g/m^2^) phenolic resin and sandwiched between orthogonally oriented untreated veneers. The glued plywood samples were cold-pressed under 0.8 MPa for 1 h at room temperature, and then hot-pressed at 130 °C under 1.2 MPa for 7 min. Plywood was stored at ambient conditions for 24 h prior to bonding strength testing.

### 2.7. Evaluation of Resin Performance

For the bonding strength test, twelve plywood specimens (25 mm × 10 mm) were cut from each panel and submersed in boiling water for 3 h according to the China National Standard GB/T 9846.3-2004, and then cooled at room temperature for 10 min before bonding strength testing. The test was performed on a CMT4000 Series Universal Testing Machine MTS\SANS (MTS, Shanghai, China) according to China National Standard GB/T 9846.3—2004.

Formaldehyde emissions were measured using the acetylacetone method according to China National Standard GB/T 18580-2017. Ten plywood specimens were cut into 15 cm × 5 cm specimens and placed in a 10-L glass desiccator that contained 300 mL of distilled water. The desiccator was kept at 20 °C for 24 h. The concentration of formaldehyde absorbed in the distilled water was determined via an ultraviolet spectrophotometer (UV) at a wavelength of 412 nm.

## 3. Results and Discussion

### 3.1. Characterization of Alkali Lignin and Demethylated Lignin Samples by FT-IR Spectroscopy

**Figure 1:**
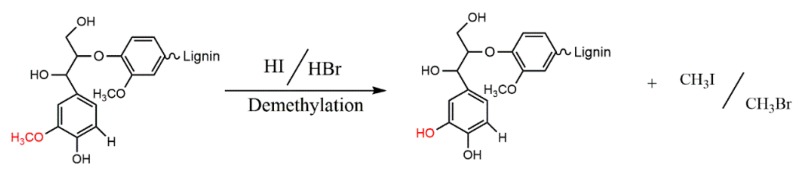
Schematic reaction process of the demethylation of lignin with halogen acids.

Nucleophilic reagents, here being two halogen acids (HI, HBr), were found to be effective reagents for increasing the concentration of free phenolic hydroxyl groups in a commercially available alkali lignin. The halogen acids, as nucleophilic reagents, combine with the methyl of a methoxyl group to form a haloalkane, thus converting methoxyl groups into phenolic hydroxyl groups (Figure 1). The aromatic ring positions ortho and para to the phenolic hydroxyls are active to combine with formaldehyde to form methylene bridges during phenolic resin synthesis. Accordingly, the through demethylation, an alkali lignin can be better suited to replace some of the phenol in the phenolic resin.

**Figure 2:**
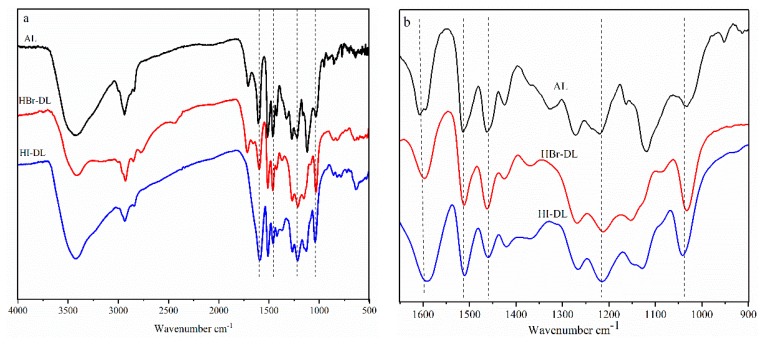
(**a**) FT-IR spectra of alkali lignin (AL) and demethylated lignins (DL) with HBr or HI as the reagent; (**b**) enlarged spectral region shows signals of particular interest.

The FT-IR spectra of the starting AL sample, and two of the DL samples, are shown in Figure 2. In very general terms, similar peak patterns are present in all of the spectra, save for minor shifting of some peaks, and as anticipated, changes in intensities for peaks of particular interest. Peaks near 1600 and 1500 cm^−1^ corresponded to the aromatic skeletal vibrations in lignin, and these bands were used as a reference peaks for relative peak intensity comparisons. The absorbance intensity of these two bands was averaged and the relative values of the other peaks were obtained by normalizing the absorbance value of the peak to the mean value of above-noted reference peaks [15].

The FT-IR spectra of the starting AL sample and two of the DL samples are shown in Figure 2. In very general terms, similar peak patterns are present in all of the spectra, save for minor shifting of some peaks, and as anticipated, changes in intensities for peaks of particular interest. Peaks near 1600 and 1500 cm^−1^ corresponded to the aromatic skeletal vibrations in lignin, and these bands were used as a reference peaks for relative peak intensity comparisons. The absorbance intensity of these two bands was averaged and the relative values of the other peaks were obtained by normalizing the absorbance value of the peak to the mean value of above-noted reference peaks [15].

All lignin samples showed a broad peak around 3400 cm^−1^ assigned to hydroxyl group stretching (Figure 2). Peaks around 1218 cm^−1^ and 1039 cm^−1^ were assigned more specifically to phenolic and primary aliphatic hydroxyl group stretching, respectively. As expected, the relative values of the phenolic hydroxyl group increased, and methoxyl group (1457 cm^−1^) decreased, in the DL samples (Table 1). The aliphatic hydroxyl group at peak 1039 cm^−1^ also increased, and could be the product of the demethylation reaction leading to the cleavage of other ether linkages in DLs, thereby forming additional phenolic hydroxyl groups, but also some aliphatic hydroxyl groups [20]. The relative values for the phenolic and primary aliphatic hydroxyl peaks, at 1218 cm^−1^ and 1039 cm^−1^, respectively, were slightly higher for the HI-DL than the HBr-DL. This is due to the lignin demethylation being a function of nucleophilic performance [21], with the iodine anion being a stronger nucleophile than the bromine anion.

### 3.2. Characterization of Alkali Lignin and Demethylated Lignins by ^1^H-NMR Spectroscopy

**Figure 3:**
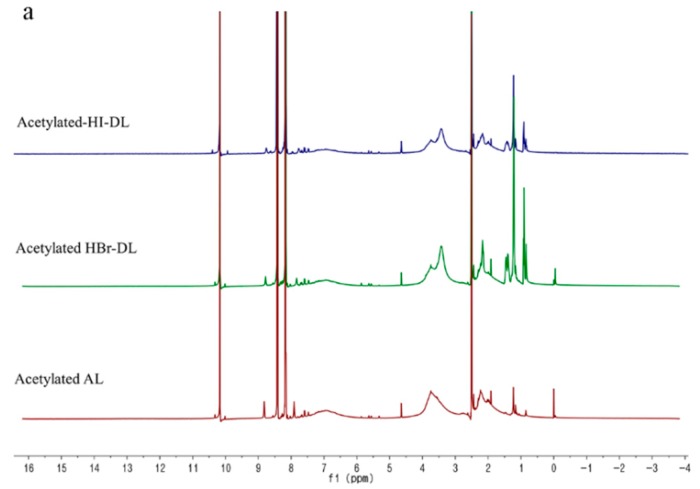

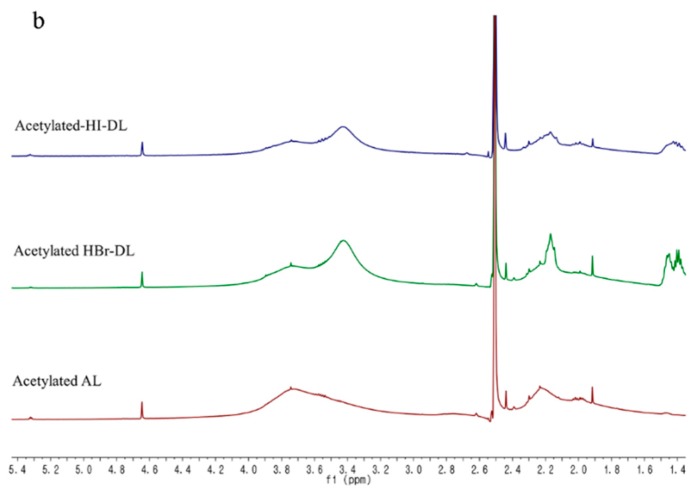
^1^H NMR spectra of (**a**) acetylated AL and demethylated lignins (DL); and (**b**) enlarged area of the methoxy and acetoxy group.

The 1H-NMR spectra of acetylated AL, and acetylated DL samples are shown in Figure 3. The proton signals observed at 10.15 ppm can be attributed to the –CHO group of p-nitrobenzaldehyde. The aromatic protons in p-nitrobenzaldehyde could be observed at 8.38 and 8.10 ppm. The aliphatic protons in lignin can be observed between 0.8 and 1.5 ppm while the aromatic protons in lignin are between 6.0 and 8.0 ppm. The aromatic protons at the C5 position in lignin are assigned to the broad signal at 6.96 ppm. The methoxyl group protons, which are closely related to the proportion of guaiacyl:syringyl units in lignin, are assigned to the broad signal at 3.82 ppm. Finally, the signals at 2.27 and 2.01 ppm were assigned to the protons of the aromatic and aliphatic acetyl groups, respectively. The methoxyl group signal (3.82 ppm) shows a decrease in intensity in both acetylated DLs compared to that of the acetylated AL. The aromatic acetyl groups (2.27 ppm) were formed by the acetylation of phenolic hydroxyl groups. In this study, we used acetoxy group measurements after the acetylation of the lignin because this method is commonly used hydroxyl group characterization method in lignin modifications.

The ^1^H-NMR spectra were integrated in order to assess changes to the AL upon demethylation. As expected, the aromatic proton signals in *p*-nitrobenzaldehyde, at 8.38 and 8.10 ppm, were similar to each other; these two peaks were used as an internal reference peaks to calculate lignin functional group content. The results for evaluating the content of specific functional groups in lignin are shown in Table 2. Relative to the internal standard, *p*-nitrobenzaldehyde, added at the same mass fraction for all lignin samples, showed the aromatic proton signals for all three acetylated lignin samples have the same molar amounts. As expected, the molar amount of phenolic hydroxyl groups increased, with values from 0.52 mmol/g to 0.67 mmol/g for HI-DL; the molar amount increased to a seemingly lesser extent (0.64 mmol/g) when HBr was used as the reagent. The higher content of phenolic hydroxyl groups in HI-DL, compared to that in HBr-DL, was consistent with the semi-quantitative FT-IR results. The amounts of aliphatic hydroxyl groups also increased. The above-mentioned increases in hydroxyl content may also be attributed to the demethylation reaction causing the cleavage of other ether linkages in lignin and forming phenolic or aliphatic hydroxyl groups [20].

Based on the FT-IR and ^1^H-NMR results, the demethylations by HBr and HI were effective in reducing the methoxyl content of an alkali lignin. Given, that significant demethylation of the alkali lignin had been achieved using halogen acids as the nucleophilic reagents, it remained to be determined whether there would be a significant increase in the performance of the PF resins prepared from the DLs; furthermore, given small differences in the chemical functionalities of the DLs produced with HBr and HI, it also remained to be determined if this would result in any detectible difference between DLPF resins produced with either HI-DL or HBr-DL.

### 3.3. HSQC Analysis of the AL and DL Samples

**Figure 4:**
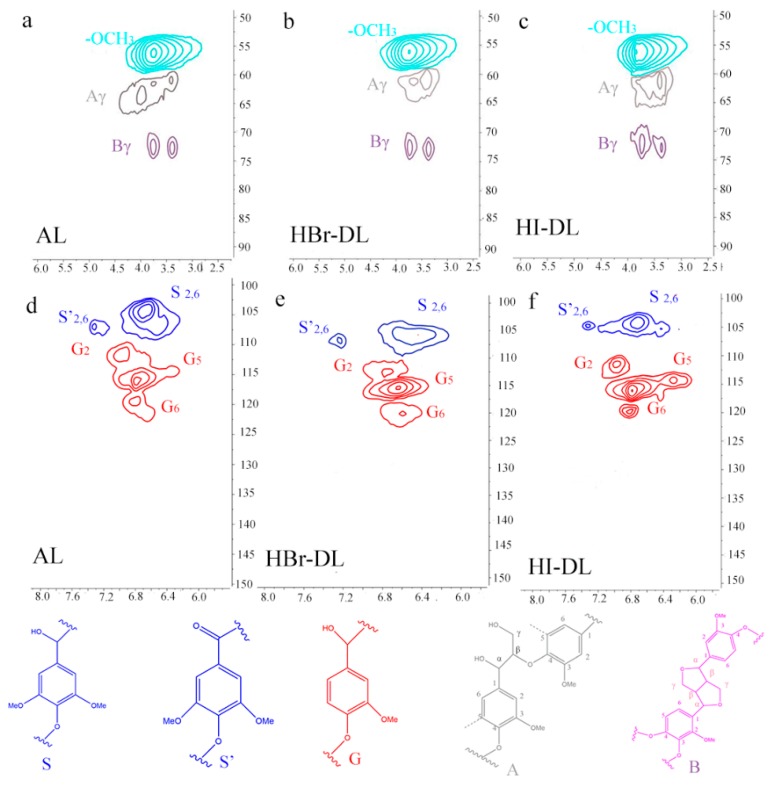
HSQC spectra of AL and DL samples: Signals for sidechain in (**a**) AL (**b**) HBr-DL (**c**) HI-DL and signals for aromatic ring in: (**d**) AL (**e**) HBr-DL (**f**) HI-DL. Assignments for ^13^C-^1^H cross-signals provided in Table 3.

The chemical transformations of the AL during demethylation were further investigated by using a 2D NMR technique, HSQC(Figure 4). Some of the main structural characteristics of AL and the DLs, including basic monomer unit type (guaiacyl, G; syringyl, S) and selected substructures, can be observed in the HSQC spectra. The corresponding ^13^C-^1^H cross-peaks were assigned (Table 3) according to the literature [22].

As an important parameter in the characterization of isolated lignins, the S/G ratio is introduced here to verify the changes to the AL upon demethylation. The relative content ratios of syringyl units to guaiacyl units in the AL and DLs are shown in Table 4. A slight increase of G units in HBr-DL (27/73) and HI-DL (21/79), as compared to the AL (38/62), was observed. This could suggest that partial demethoxylation occurred on S units leading to a relative increase in the content of G units; alternatively, as the demethoxylations progressed, that for the S units was more complete than that for the G units, hence giving lower S/G ratios. The G unit has a free C_5_ position (ortho to the phenolic hydroxyl) on the aromatic ring; on the other hand, in the S unit, both C_3_ and C_5_ positions have methoxy groups. From this point of view, a lower S/G ratio coincides with and increases the phenolic hydroxyl units in lignin, and thus increases the active ortho positions for phenolic resin synthesis. Specifically, it improves the ability of phenolic hydroxyl ortho-position of lignin to form a methylene bridge with formaldehyde. Altogether, the HSQC data, along with the FT-IR and ^1^H- NMR data show that the AL was demethylated, improving its potential to partially replace phenol to prepare phenolic resins.

### 3.4. Characterization of PF, ALPF, and DLPF Resins

**Figure 5:**
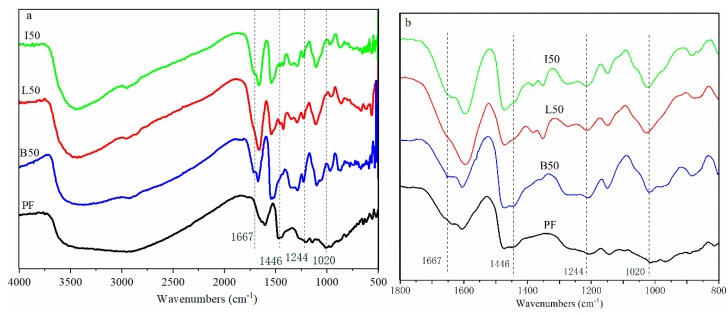
(**a**) FT-IR spectra of PF, ALPF (L50), and DLPF (I50, B50) resins with 50% lignin substitution; (**b**) enlarged spectral region shows signals of particular interest.

As a preliminary assessment of chemical functionality differences between the ALPF and DLPF resins, FT-IR spectra were collected from each of the resin samples prepared using the AL and DL samples. Results showed the spectra for the ALPF and DLPF resins were similar, and thus while differences could be observed in the FT-IR spectra of the AL and DL lignins themselves, any such differences were generally masked when these lignins were incorporated into the PF resins. Compared to the PF resin itself, the ALPF and DLPF resins showed some bands typical of lignin. For example, the lignin band at 1667 cm^−1^, attributed to unconjugated carbonyl groups, and that at 1446 cm^−1^, attributed to lignin- and phenol-derived aromatic rings, were observed in the lignin-containing resins. Similarly, all ALPF and DLPF samples showed higher absorption values at 1020 cm^−1^ than the PF, which was assigned to the C–O stretching vibration of aliphatic C–OH, aliphatic C–O(Ar) and methylol C–OH groups in lignin [23] (Figure 5); other lignin bands included 1130 cm^−1^ (stretching vibration of ether linkages [24]) and 1210 cm^−1^ (guaiacyl and syringyl ring C–O stretching vibrations [25]). Altogether, the FT-IR spectra were consistent with a PF resins alone, or those in which a lignin, either modified or not, was added.

**Figure 6:**
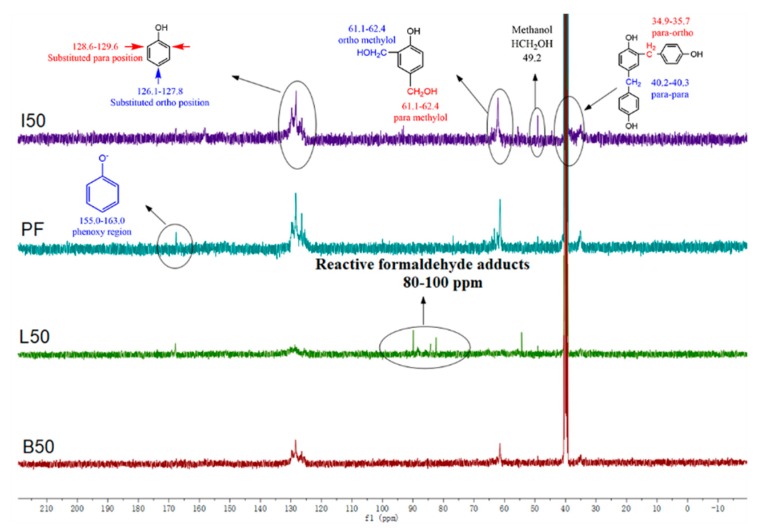
^13^C-NMR spectra of PF, ALPF(L50), and DLPF (I50, B50) resins with 50% lignin substitution.

An assessment of the effect of the AL and DL substitutions for phenol on the chemical functionality of PF resins was also carried out using the liquid-state ^13^C-NMR. As expected, the polymeric nature of the resins afforded spectra (Figure 6) for which the signal-to-noise ratio was low; however, in general terms, the ^13^C-NMR showed signals at 40.2–40.3 ppm (para-para methylene bridges) and 34.9–35.7 ppm (para-ortho methylene bridges) [26] in PF resins. The presence of methanol formed during resin preparation process from the Canizzaro reaction of formaldehyde was shown by the signal at 49.2 ppm. Methylol groups on the ortho and para positions to the phenolic hydroxyl groups were shown by the signals at 61.1–62.4 ppm and 63.3–64.5 ppm, respectively. The dominant signals at 128.6–129.6 ppm and 126.1–127.8 ppm were assigned to the substituted ortho and para positions on the aromatic rings, respectively. It is worth mentioning that signals at chemical shifts from 80–100 ppm, readily visible in the ALPF sample, were reactive formaldehyde adducts [27].

Comparing between the ALPF, DLPF, and PF resins, it appears that the para-para methylene bridges (40.2–40.3 ppm) were much higher than the para-ortho methylene bridges (34.9–35.7 ppm). The integral intensity of methylol groups at 63.3–64.5 ppm and 61.1–62.4 ppm in ALPF resins were lower than the other three resins, and is attributed to the lower reactivity of the AL, relative to the DLs, and phenol. In addition, the PF, BLPF, and ILPF resins also have higher integral intensity at 128.6–129.6 ppm and 126.1–127.8 ppm, assigned to the substituted ortho and para carbons on the aromatic rings, respectively. This is due to the improved reactivity of DLs by demethylation. Weak peaks in the spectra of ALPF (80 ppm to 100 ppm), assigned to the active formaldehyde adducts, can be attributed to the lower reactivity of the AL.

### 3.5. Curing and Mechanical Properties of PF, ALPF and DLPF Resins

**Figure 7:**
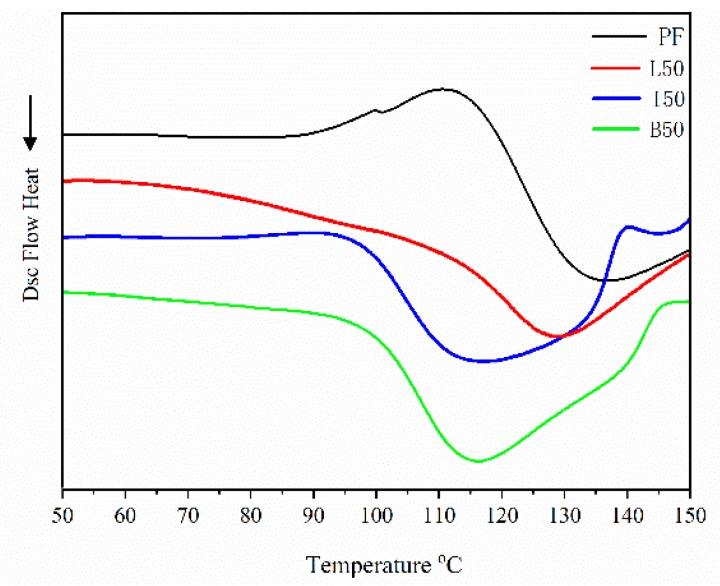
DSC analysis of PF, ALPF(L50), DLPF (I50, B50) resins with 50% lignin substitution.

Amorphous polymers such as lignin undergo a transition from a glassy state to a rubbery state at a particular temperature, which is referred to as the glass transition temperature or *T*_g_. This is one of the significant characteristics of a polymer, as it affects its application and processing [28]. Thus, in addition to attempting to discern differences in chemical functionality between the PF, ALPF, and DLPF resins, we explored their curing behavior using differential scanning calorimetry (DSC). The DSC thermograms of PF, ALPF and DLPF resins are shown in Figure 7, and the related measures of peak temperature (*T*_p_), onset temperature (*T*_o_), Δ*T* (*T*_p_−*T*_o_), and *T*_g_ are shown in Table 5. The value for Δ*T* indicates the difference in peak temperature and onset temperature of the curing reaction in the endotherm. Since the rate of heating is uniform in all the cases, with the same amount of sample size, this difference in temperature is indicative of relative curing times [29]. The results showed the longest Δ*T* value, or curing time for PF resin. Faster curing times, due to the incorporation of lignin, have been reported in the literature [29]. As is to be expected, the values for *T*_g_ trend with the curing times. Among the resins, the thermograms for the DLPFs were quite similar, but both were different than that for the ALPF. This provided evidence that lignin demethylations occurred to an extent that was sufficient to impact the thermal properties among lignin-containing PF resins. Differences between the DLPFs and the PF were expected, and demonstrate that DLs are not direct drop-in replacements for phenol; however, it remained to be determined whether the substitution of phenol with DLs impacted performance, specifically as an adhesive.

**Figure 8:**
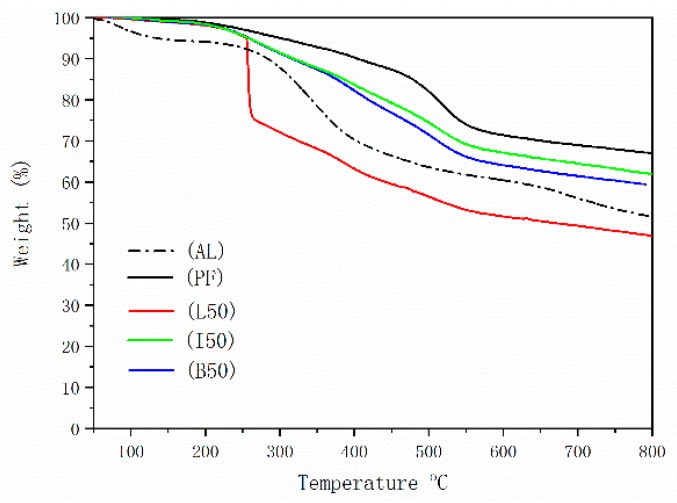
TGA analysis of Alkali lignin, PF, ALPF(L50), DLPF (I50, B50) resins with 50% lignin substitution.

Thermogravimetric analysis (TGA) was also performed to assess the thermal stability and decomposition of the PF, ALPF, DLPF resin samples. As can be seen in the thermograms (Figure 8), AL shows a typical thermal decomposition process of an isolated lignin. In the initial degradation stage, occurring between 50 °C and 130 °C, the loss of mass can be attributed in part to the evaporation of water; mass continued until final amount of non-volatile residue of 51.6% remaining at 800 °C. As expected, the thermograms for the resins were distinctly different than the AL. The thermal degradation of PF resins can be divided into three stages, those being post curing, thermal reforming, and ring stripping [30]. In the first two stages, the mass loss can be attributed to the evaporation of water from condensation reactions of methylol groups in the first stage, and from condensation reactions of methylene and phenolic OHs, as well as between two hydroxyl functional groups, in the second stage. In the third stage, the mass loss can be attributed to the evolution of carbon monoxide and methane, through the thermal degradation of methylene bridge [31]. Resins B50 and I50 each showed a similar thermal decomposition curve to the PF resin. Unique to the L50 was a sharp weight loss (ca. 20%) from 250 to 265 °C; this was attributed to the phenolic hydroxyl content of the AL, resulting in poor crosslinking within the phenolic resin, and thus resulting in lower thermal stability. From the perspective of amounts final residue at 800 °C, that for I50 (62%) and B50 (59%) were higher than that for L50 (47%). This demonstrated that the reactivity of the lignin through demethylation is improved, with higher phenolic resin crosslinking resulting in higher thermal stability. Consistent with this observation is that the final residue for I50 (62%) was slightly higher than that for B50 (59%), also due to the different levels of reactivity for the two DLs.

**Figure 9:**
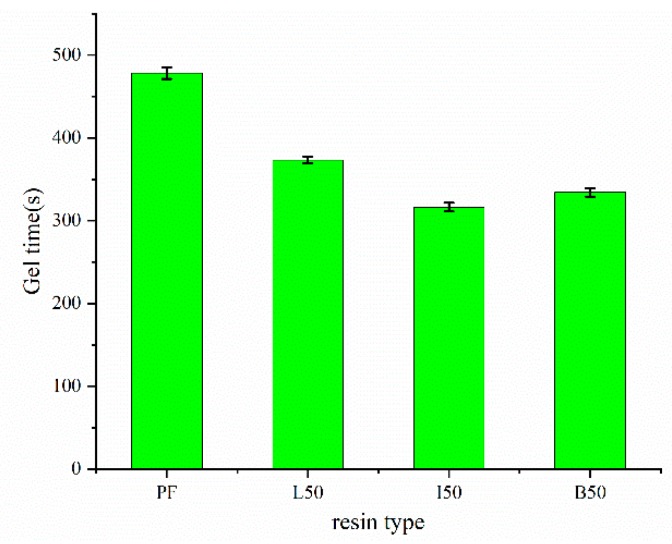
Gel time of PF, ALPF (L50), and DLPF (I50, B50) resins with 50% lignin substitution.

Figure 9 shows the gel times for the PF, ALPF and DLPF resins. Gel time is generally defined as the time required for the pre-polymer to transform from a disorganized liquid into a 3-dimensional macromolecular structure under specified conditions. Similar to Δ*T* discussed above, gel time can also be used to assess the curing rate and reactivity of phenolic resins. Results showed the ALPF and DLPF resins exhibited shorter gel time and faster curing rates than the PF resin. This was attributed to the substitution of high molecular weight polymers, the AL and DLs, for phenol; the combination of lignin and the macromolecular structure of a phenolic resin has been shown to result in a reduced curing time [32]. These results parallel the DSC data. Comparing the HBr-DLPF to HI-DLPF resins, a slightly faster curing time for the latter was observed. This result can be attributed to the higher content of phenolic hydroxyls in the HI-DL relative to HBr-DL.

**Figure 10:**
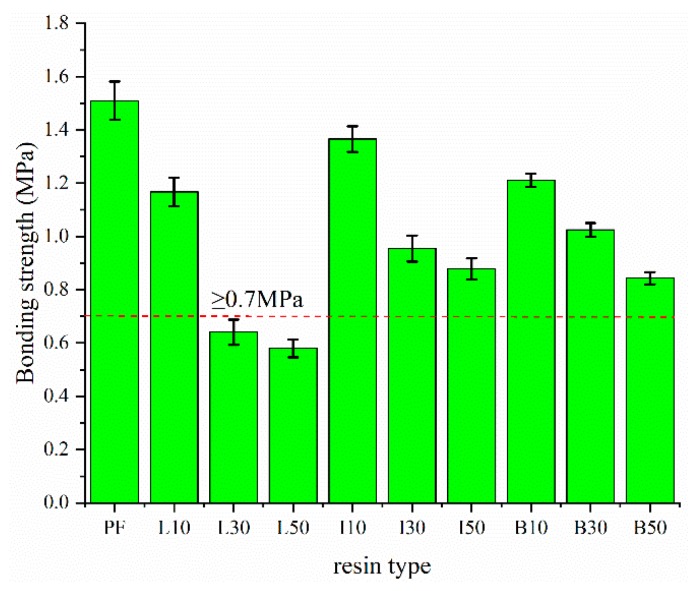
Bonding strength of PF, ALPF, DLPF resins at different lignin substitutions.

An objective of the present study was to demethylate a lignin, and thereby improve its reactivity in a PF resin. By extension, any improvement in reactivity should manifest itself in improved performance of the resultant DLPFs in the bonding of wood. A common test used to evaluate thermoset adhesives, such as phenolic resins, is bonding strength. Plywood panels were prepared using the PF, ALPF and DLPF resins at a 50% lignin substitution for phenol; anticipating that such a high level of lignin substitution would have a detrimental impact on bonding strength, additional panels were fabricated at 10% and 30% lignin substitutions. As expected, the best performance was shown for the PF resin, with a bonding strength of 1.51 MPa (Figure 10). For each of the lignin-containing resins, higher lignin substitutions resulted in lower bonding strengths. Nevertheless, the bonding strength of with both DLPF resins, at all lignin substitutions, still met the Chinese national standard (GB/T 9846-2015) for exterior-grade plywood panels (≥ 0.7 MPa). Only the plywood bonded with ALPF resin did not satisfy national standard when the lignin substitution was 30% or higher. Through the modification of the lignin by demethylation, at DL substitutions as high as of 50%, the bonding strength was 0.88 MPa (I50) and 0.85 MPa (B50), still meeting the standard for exterior-grade plywood.

### 3.6. Formaldehyde Emission

**Figure 11:**
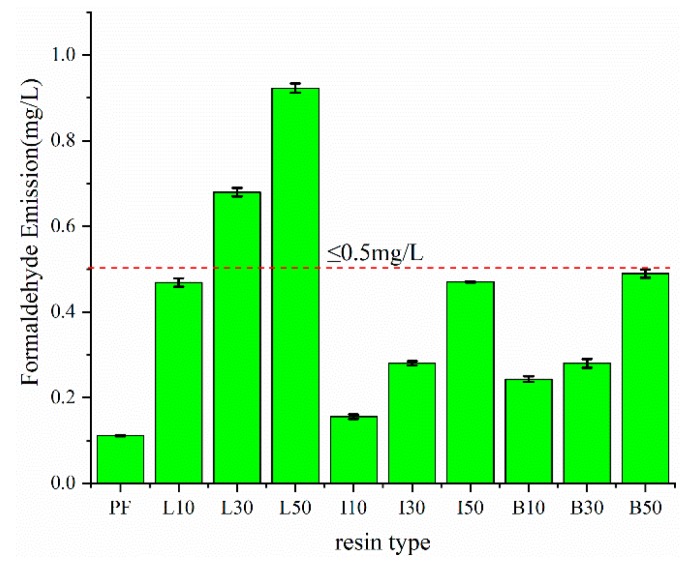
Formaldehyde emission of PF, ALPF, and DLPF resins at different lignin substitutions.

Similar to the bond strength data, the formaldehyde emission data also trended with the lignin substitutions, but here, formaldehyde emissions increased with increasing lignin substitution. Since the increasing levels of lignin, either AL or DL, would likely result in fewer methylene bridges in the resin, there is a greater likelihood of residual formaldehyde. Intuitively, since the modification of the lignin by demethylation increased reactivity, and bonding strength, we predicted that said lignin modification would also result in lower formaldehyde emissions. Results for the plywood bonded with DLPF and PF resins (Figure 11) all showed lower formaldehyde emissions and were less than *E*_0_ grade (0.5 mg/L) for the Chinese national standard (GB/T 9846-2015). On the other hand, plywood bonded with ALPF resin exceeded the standard when the lignin substitution was 30% or higher. Regarding the two different demethylation reagents, HI and HBr, the former showed greater demethylation, greater HI-DL reactivity and greater HI-DLPF bonding. All of these results are consistent with lower formaldehyde emissions in the HI-DLPF resins, relative to the HBr-DLPF resins.

Altogether, demethylated lignins were produced by the modification of a commercially available alkali lignin through reactions with halogen acids. These modified lignins were then used for the preparation of lignin-based phenolic resins. This demonstrates how chemical modification strategies applied to natural polymers can be used to convert them into replacements for petroleum-derived monomers currently used in the preparation of commodity polymers. Halogen acids were undoubtedly an efficient demethylation reagent for lignin due to their nucleophilicity. This may promote more natural products with insufficient utilization rates to be more fully utilized in industrial production, and produce more economic benefits; however, the application of halogen acids in industrial production of demethylated lignins will require the development of strategies to recycle the halogen acids to reduce wastewater treatment costs.

## 4. Conclusions

Lignin demethylation with halogen acids (HI, HBr) was used to increase the hydroxyl content of lignin with the intent to improve lignin reactivity, and ultimately, the performance of the resultant lignin-containing PF resins. The content of phenolic hydroxyl groups increased for both DLs, but to a greater extent for that demethylated with HI. Aliphatic hydroxyl group contents increased to the same extent for both DLs. The higher degree of demethylation with HI is attributed to its stronger nucleophilicity compared to HBr. Regarding the PF resins prepared by substituting AL, or DLs, for phenol, the DLs showed better performance in terms of greater reactivity, greater bonding strength of plywood panels, and lower formaldehyde emissions. Altogether, lignin demethylation with a halogen acids can afford a modified lignin that can replace up to 50% of phenol in PF resins and still meet performance standards for exterior-grade plywood panels. The stronger nucleophilicity of HI, compared to HBr, impacted the degree of lignin demethylation and carried through to measurable differences the thermal properties and performance of lignin-containing PF resins.

## Figures and Tables

**Table 1:** FT-IR peak assignments and relative absorption values of AL and DL samples.

Lignin	1592 Ar–H	1509 Ar–H	1457 OCH_3_	1218 Ar–OH	1039 Al–OH
AL	1	0.93	1.57	0.95	0.79
HBr–DL	1	1.145	1.29	1.34	1.09
HI–DL	1	1.15	1.23	1.35	1.20

**Table 2:** Semi-quantitative comparison of phenolic and aliphatic hydroxyl groups after demethylation based on ^1^H-NMR analysis of acetylated lignins.

	Proton Arrangement	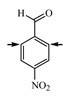	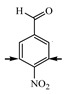	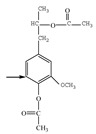	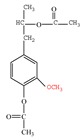	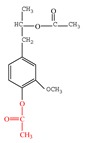	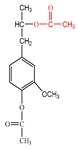
	Chemical Shift (ppm)	8.38	8.10	6.95	3.82	2.27	2.03
**Acetylated AL**	Molar Amount of Functional Groups in 1 g Lignin (mmol/g)	7.02		6.38	1.54	0.52	0.37
**Acetylated HI-DL**		7.02		5.34	1.10	0.67	0.47
**Acetylated HBr-DL**		7.02		6.11	1.18	0.64	0.47

**Table 3:** Assignments of main AL and DLs ^13^C-^1^H cross-signals in the HSQC spectra.

Label	δC/δH (ppm)	Assignment
–OCH_3_	55.6/3.74	C−H in methoxyls
A_γ_	59.5–59.7/3.40–3.63	C_γ_−H_γ_ in β-O-4′ substructures (A)
B_γ_	71.3/3.99 and 71.3/4.37	C_γ_−H_γ_ in resinol substructures (B)
S_2,6_	103.7/6.71	C_2,6_−H_2,6_ in etherified syringyl units (S)
S′_2,6_	106.3/7.25	C_2,6_−H_2,6_ in oxidized (C_α_=O) syringyl units (S′)
G_2_	112.9/6.98	C_2_−H_2_ in guaiacyl units (G)
G_5_	115.0/6.77	C_2_−H_2_ in guaiacyl units (G)
G_6_	119.0/6.80	C_6_−H_6_ in guaiacyl units (G)

**Table 4:** Determination of S/G ratios for the AL and DLs by quantifying monomer unit type HSQC signals.

Samples	AL	HBr-DL	HI-DL
**S/G ratio**	38/62	27/73	21/79

**Table 5:** Thermal properties of PF, ALPF and DLPF resins at 50% lignin substitution.

Resin	DSC Analysis and Endothermic Event			
	*T*_p_ (°C)	*T*_o_ (°C)	Δ*T* (°C)	*T*_g_ (°C)
PF	135	110	25	123
L50	128	106	22	117
I50	115	100	15	107
B50	116	99	17	108
